# *PTGER3* and *MMP-2* play potential roles in diabetic nephropathy via competing endogenous RNA mechanisms

**DOI:** 10.1186/s12882-020-02194-w

**Published:** 2021-01-12

**Authors:** Yue Yu, Yuan-Yuan Jia, Meng Wang, Lin Mu, Hong-Jun Li

**Affiliations:** 1 Department of Endocrinology, China-Japan Union Hospital of Jilin University, Changchun, 130033 Jilin Province China; 2 China-Japan Union Hospital of Jilin University, Changchun, 130033 Jilin Province China; 3 Center of Reproductive Medicine, Center of Prenatal Diagnosis, the First Hospital of Jilin University, Changchun, 130021 Jilin Province People’s Republic of China; 4 Department of Radiology, The First Hospital of Jilin University, Changchun, 130021 Jilin Province People’s Republic of China; 5 Health Management Medical Center, China-Japan Union Hospital of Jilin University, 126 Xiantai Street, Changchun, 130033 Jilin Province China

**Keywords:** Diabetic nephropathy, Microarray, microRNAs, Type 2 diabetes mellitus, Molecular biology

## Abstract

**Background:**

Diabetic nephropathy (DN) is a primary complication of diabetes mellitus (DM). The pathology of DN is still vague, and diagnostic accuracy is not enough. This study was performed to identify miRNAs and genes that have possibilities of being used as therapeutic targets for DN in type 2 DM.

**Methods:**

Human miRNA data GSE51674 and gene data GSE111154 were downloaded from the Gene Expression Omnibus database. Differentially expressed genes (DEGs) and miRNAs (DEmiRNAs) in the kidney between control and DN patients were screened out. The competing endogenous RNA (ceRNA) network was constructed, and key lncRNA-miRNA-mRNA pairs were selected accordingly. Potential drugs targeting DEGs were screened out and validated using PCR analysis.

**Results:**

Totally, 83 DEmiRNAs and 293 DEGs were identified in GSE51674 and GSE111154, respectively. Thirteen of the top 20 DEmiRNAs (10 up and 10 down) targeted to 47 DEGs. In the ceRNA network, *RP11-363E7.4*/*TTN-AS1*/*HOTAIRM1*-*hsa-miR-106b-5p*-*PTGER3* and *LINC00960*-*hsa-miR-1237-3p*-*MMP-2* interaction pairs were identified as the key ceRNA network. Interestingly, *PTGER3* and *hsa-miR-1237-3p* were downregulated, and *MMP*-2 and *hsa-miR-106b-5p* were upregulated in the kidney of patients with DN compared with normal controls, respectively. *PTGER3* and *MMP*-2 were targeted by drugs including iloprost, treprostinil, or captopril, and the deregulation of the two genes was confirmed in the plasma samples from patients with DN as compared with controls.

**Conclusions:**

We speculated that the *RP11-363E7.4*/*TTN-AS1*/*HOTAIRM1*-*hsa-miR-106b-5p*-*PTGER3* and *LINC00960*-*hsa-miR-1237-3p*-*MMP-2* networks were associated with diabetic renal injury.

## Highlights

1. Renal *PTGER3* and *miR-1237-3p* were reduced in diabetic nephropathy (DN).
2. *MMP-2* and *miR-106b-5p* were increased in the kidney of patients with DN.
3. *PTGER3* reduction and *MMP*-2 upregulation may protect renal injury from diabetes.

## Background

Diabetes mellitus (DM) is a metabolic disorder that affects 1 in 11 adults aged between 20 and 79 years old in 2015 [1–3]. The global estimated prevalence of DM is expected to rise to 578 million by 2030 and ~ 700 million by 2045 [4, 5]. People with DM are at high risk of developing serious problems and life-threatening health complications, including diabetic nephropathy (DN), retinopathy, cardiovascular disease, and stroke [1, 6–8]. DN is the primary cause of end-stage renal failure and chronic kidney disease [6]. It is a major chronic complication that affects over 40% of DM and ~ 10% of type 2 DM died of renal failure globally [1, 3, 9, 10].

The pathophysiology leading to DN and resultant renal failure from DM consists of hypertension, altered composition and proliferation, and sclerosis in glomerular [10]. Accordingly, the estimated glomerular filtration rate (eGFR), albuminuria, and hypertension are the clinical manifestations of DN [3]. However, the clinical usage of them could not make an accurate and definite diagnosis of DN and the degree of kidney damage [3]. Herein, DN is often confirmed during postmortem examination because of the lack of definitive clinical findings. Theoretically, the progression and complications of DN could be blocked or slowed down by early-stage interventions [10–12]. A major obstacle for that is the lack of accurate biomarkers identifying patients who at high risk of DN. To fulfill this gap, experiments are being carried out to find effective methods for the accurate and preoperative diagnosis of DN [13].

Much evidence has shown that genetic factors are the major predispositions to type 2 DM and DN [1, 10]. Many non-coding RNAs and genes like transforming growth factor (TGF) β, nephrin, angiotensin-converting enzyme-2, interleukin-6 (IL-6), and tumor necrosis factor (TNF)-α have been identified to be associated with the development, progression, and prognosis of DN [9, 12–14]. These genetic factors modulate extracellular matrix structure as well as the oxidative, fibrotic, and inflammatory responses in the kidney, tubular, and glomerular [12, 15, 16]. Nevertheless, no single treatment targeting the above factors has been able to reverse or mitigate DN progression, or reduce DN-associated mortality [14]. Therefore, additional factors that have therapeutic targeting possibilities in DN are in urgent need.

This study was performed to identify the genes and miRNAs that were differentially expressed in the kidney of patients with DN. Integrated bioinformatics analysis was performed for screening the miRNAs and genes in DN in type 2 DM. Moreover, the possibility of using them as therapeutic targets would be discussed.

## Methods

### Microarray data

The human miRNA microarray dataset GSE51674 and gene expression dataset GSE111154 were downloaded from the National Center for Biotechnology Information (NCBI) Gene Expression Omnibus (GEO, http://www.ncbi.nlm.nih.gov/geo/). GSE51674 (GPL10656 Agilent-029297 Human miRNA Microarray v14 Rev.2; miRNA ID version) consisted of 10 kidney samples from 4 controls (normal kidney from patients underwent kidney biopsy) and 6 DN patients (kidney tissues from patients with type 2 DM for 15 ± 7 years and biopsy-proven DN) [6]. GSE111154 (GPL17586 [HTA-2_0] Affymetrix Human Transcriptome Array 2.0, transcript (gene) version) consisted of 8 kidney cortical tissue samples from 4 non-diabetic controls and 4 DN patients (type 2 DM with early DN) [3].

### Data processing

The raw data in GSE51674 and GSE111154 datasets were downloaded and processed using the Limma package [17] and Oligo package [18], respectively. Data processing included background correction, normalization, and expression calculation. The probes in GSE51674 and GSE111154 that mapped to human miRNAs and mRNAs, respectively, were retained and used for further analysis. When multiple probes mapped to one gene symbol, the averaged expression level of probes was calculated and regarded as the gene expression level of that gene or miRNA. The probes that did not map to genes or human miRNAs were removed.

### Differential expression analysis

The differentially expressed genes (DEGs) and miRNAs (DEmiRNAs) between control and disease samples were identified using the paired t-test in the Limma package [17]. Significant DEGs were identified according to the criteria of *p* value < 0.05 and |log_2_(fold change, FC)| > 0.585, and significant DEmiRNAs were screened out according to stricter criteria of |log_2_FC| > 2 and adjusted (BH correction) p value < 0.05.

### Construction of protein-protein interaction (PPI) network

The protein-protein interaction (PPI) pairs among the products of DEGs were screened in the STRING (Version 10.0, http://www.string-db.org/) [19]. The interaction pairs with a score of higher than 0.4 (medium confidence) were retained and used for the construction of the PPI network. Cytoscape (version 3.2.0, http://www.cytoscape.org/) was employed for the construction of the PPI network. Dominant modules in the PPI network were identified using the MCODE plugin (version 1.4.2, http://apps.cytoscape.org/apps/MCODE) that was provided by Cytoscape. The DEGs in modules were employed for the enrichment analysis.

### Functional enrichment analysis

The functional enrichment analysis was performed separately for the DEGs. The clusterprofiler in R was used for enriching the Gene Ontology biological processes and Kyoto Encyclopedia of Genes and Genomes (KEGG) pathways. Significant enrichment was identified with the thresholds of adjusted (BH correction) *p* value< 0.05 and gene number count ≥2.

### Prediction of miRNA-mRNA target

To investigate the correlation between the DEmiRNAs and DEGs in DN, the predictive targets of DEmiRNAs were screened from the DEG list and the miRNA-mRNA regulatory network was constructed accordingly. The targets that were collected by at least 6 of the 7 databases (miRWalk, miRanda, miRDB, miRMap, Pictar2, RNA22, and Targetscan) were regarded as potential targets in our study. Then, the miRNA-mRNA target pairs were obtained and used for the construction of the miRNA-mRNA regulatory network using Cytoscape.

### Prediction of lncRNAs that sponge DEmiRNAs

To investigate the competing endogenous RNA (ceRNA) involving lncRNAs, DEGs, and DEmiRNAs in DN, the potential lncRNA-miRNA pairs were identified in the DIANA-LncBase V2 [20]. The lncRNAs that tightly interacted with the DEmiRNAs (score ≥ 0.99) in the kidney were selected. The miRNA-mRNA and lncRNA-miRNA interaction pairs that are linked by the same miRNAs were retained and used for the construction of the lncRNA-miRNA-mRNA (ceRNA) network. Cytoscape was used for the construction of the ceRNA network.

### Drug-gene interaction network

Drug-Gene Interaction database (DGIdb) provides information on the interactions between drugs and genes, which might be of great value for settling potential therapeutic strategy that targets the key genes. DGIdb was employed to extract the drugs targeting the DEGs in the above ceRNA network. Drugs that have been admitted by the Food and Drug Administration (FDA) were reserved and used to construct the drug-gene interaction network.

### Patients and ethical statement

The peripheral blood samples were collected from three patients with DN and three healthy controls from the Department of Endocrinology, China-Japan Union Hospital of Jilin University, Changchun, China, in Sept 2020. Approval was obtained from the Ethics Committee of China-Japan Union Hospital of Jilin University, and written informed consent was obtained from all participants before the collection of blood samples. All the samples were stored at 4 °C before RNA extraction.

### RNA isolation and PCR amplification

The total RNA was isolated from the plasma samples using RNAiso Plus (Trizol; TaKaRa, Tokyo, Japan; #9109). Reverse transcription into cDNA was performed using a PrimeScript™ II 1st Strand cDNA Synthesis Kit (TaKaRa, #6210A). The real-time quantitative PCR amplification was performed using the Power SYBR Green PCR Master Mix (Thermo Fisher Scientific, Applied Biosystems, #4367659). The reaction conditions were: 50 °C for 2 min; 95 °C for 2 min; 40 cycles of 95 °C for 15 s, 60 °C for 60 s. The primers used for the amplification of *PTGER3* were: forward 5′- CGCCTCAACCACTCCTACAC-3′, reverse 5′- GACACCGATCCGCAATCCTC-3′, and the primers used for the amplification of *MMP-2* were: forward 5′- TACAGGATCATTGGCTACACACC-3′, and reverse 5′- GGTCACATCGCTCCAGACT-3′. The primers used for the amplification of the internal reference gene *GAPDH* were: forward 5′- TGACAACTTTGGTATCGTGGAAGG-3′, and reverse 5′- AGGCAGGGATGATGTTCTGGAGAG-3′. The relative expression levels of *MMP-2* and *PTGER3* were calculated using the 2^-△△Ct^ methods.

### Statistical analysis

The expression levels of the *MMP-2* and *PTGER3* genes were expressed as mean ± standard deviation. The differences in the expression levels of *MMP-2* and *PTGER3* were analyzed using the t-test in Graphpad Prism 5 (Graphpad Software, San Diego, CA). *P* < 0.05 was set as the threshold for the significant difference.

## Results

### Identification of DEmiRNAs and DEGs

**Fig. 1:**
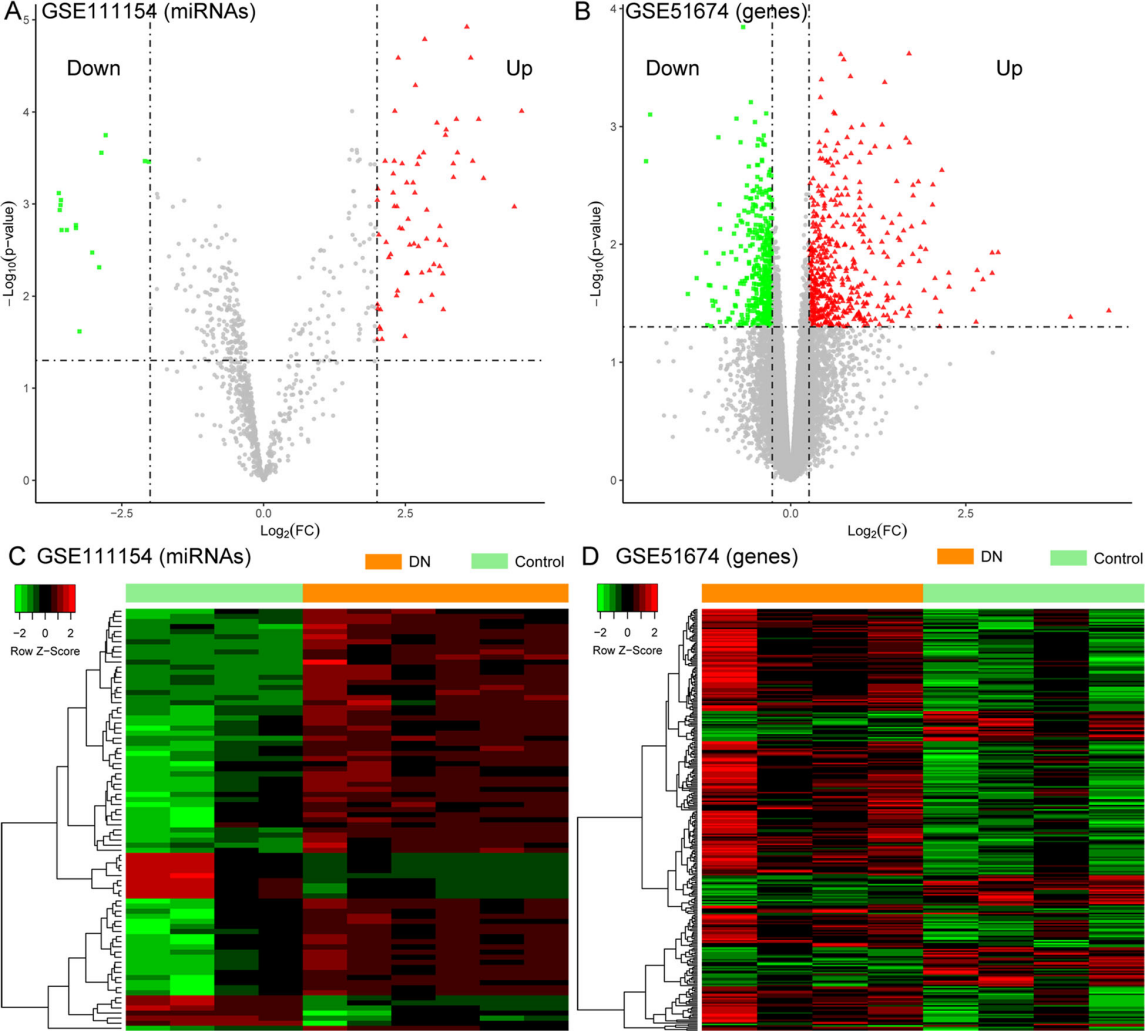
The summary of differentially expressed miRNAs (DEmiRNAs) and genes (DEGs) in diabetic nephropathy (DN) samples versus control samples. **a** and **b**, the Volcano plot of the DEmiRNAs (GSE51674) and DEGs (GSE111154) between DN and control samples, respectively. **c** and **d**, the heatmap presenting the expression profiles of the DEmiRNAs (GSE51674) and DEGs (GSE111154) between DN and control samples, respectively

After data normalization, a total of 83 DEmiRNAs (|log_2_FC| > 2 and adjusted *p* < 0.05) and 293 DEGs (p < 0.05 and |log_2_FC| > 0.585) were identified in the GSE51674 dataset and the GSE111154 dataset, respectively (Fig. 1a and b). The sample clusterings based on these DEmiRNAs and DEGs are presented in Fig. 1c and d, respectively. The heatmaps indicated that expression profiles of these genes were different between the DN and control samples.

### Analysis of the PPI network

**Fig. 2:**
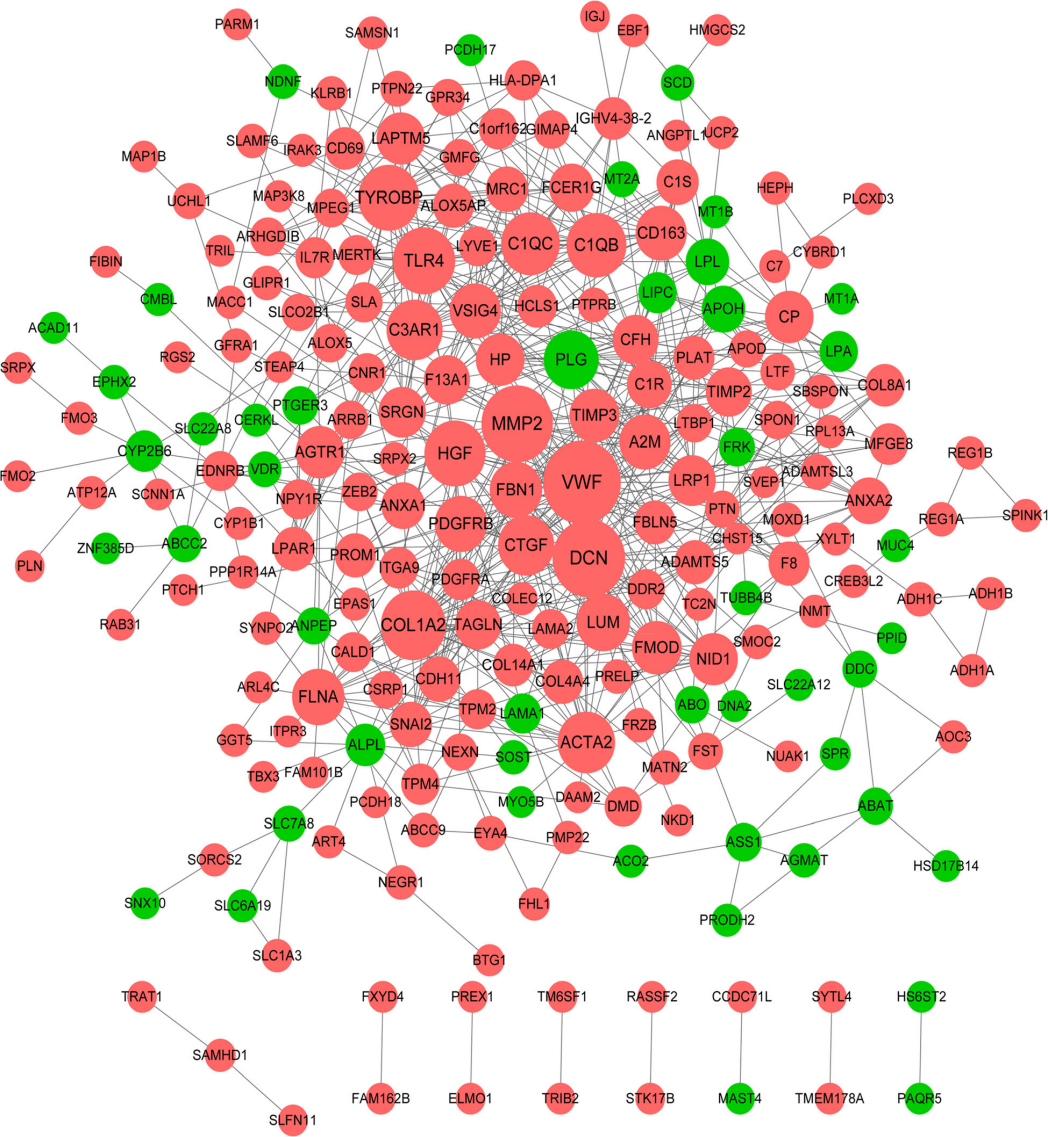
The protein interaction network of the differentially expressed genes (DEGs). Orange and green circles are the upregulated and downregulated DEGs, respectively. The node size indicates the coordinated interaction degree. The higher the interaction degree, the larger the node size is

**Fig. 3:**
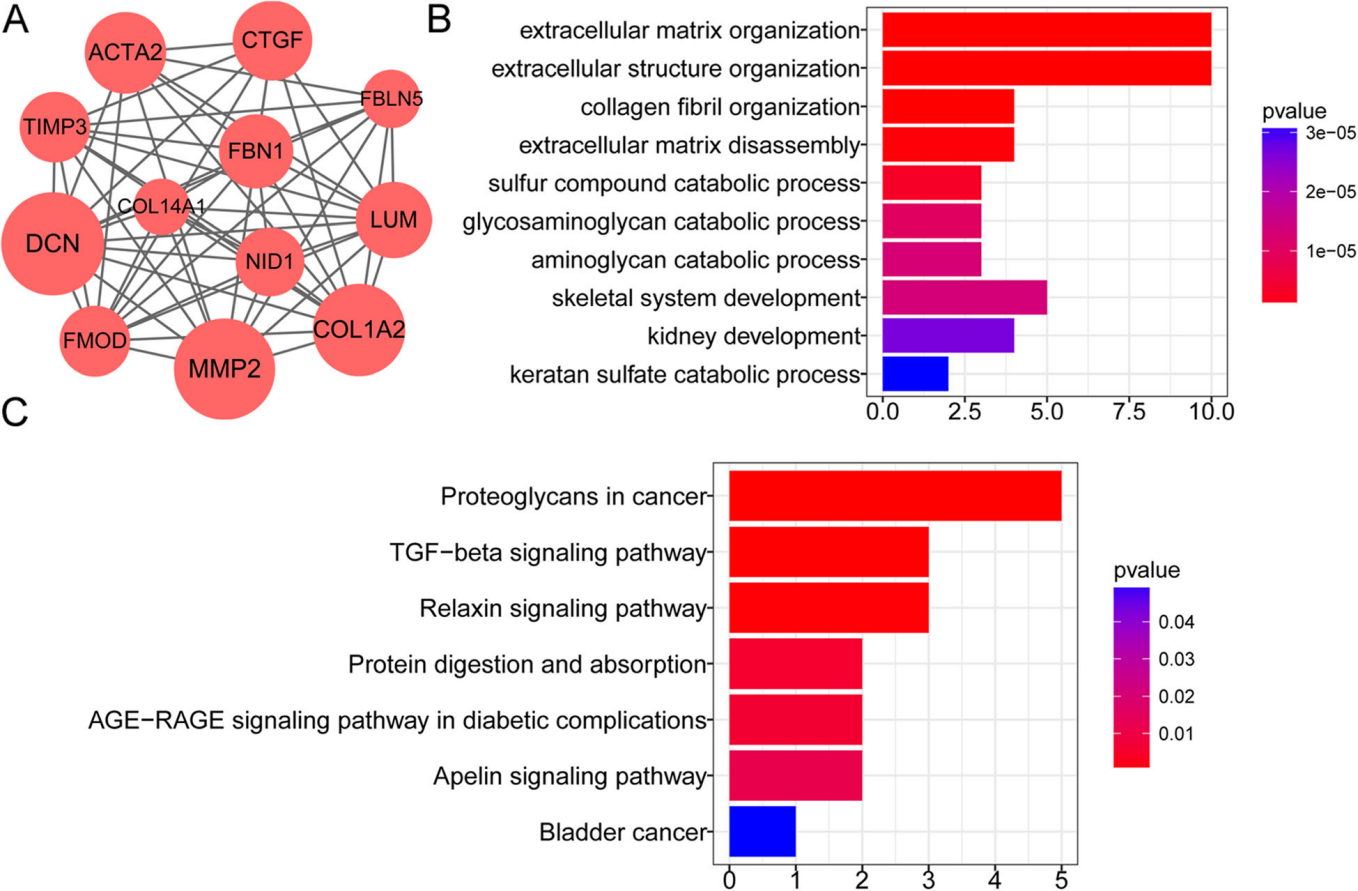
The enrichment analysis of the 12-gene module. **a**, the dominant module that consists of 12 upregulated genes. The node size indicates the coordinated interaction degree. **b** and **c**, the biological processes and KEGG pathways that significantly enrich the genes in the module

Based on the predicted protein interactions in the STRING database, the PPI network that consisted of 228 DEGs and 732 interaction pairs was constructed (Fig. 2). A dominant module with a score of 10.364 was identified (Fig. 3a). This module was composed of 12 products of upregulated genes and 57 interaction pairs. In this module, *MMP*-2, *COL1A2*, *ACTA2*, and *CTGF* had high interaction degrees of 34, 29, 23, and 22, respectively. These DEGs in the module were involved in biological processes like ‘extracellular matrix organization’, ‘extracellular matrix disassembly’, ‘sulfur compound catabolic process’, ‘glycosaminoglycan catabolic process’, and ‘kidney development’ (Fig. 3b), and KEGG pathways like ‘TGF-beta signaling pathway’, ‘Relaxin signaling pathway’, ‘AGE-RAGE signaling pathway in diabetic complications’, and ‘Apelin signaling pathway’ (Fig. 3c).

### Construction of the miRNA-mRNA regulatory network

**Fig. 4:**
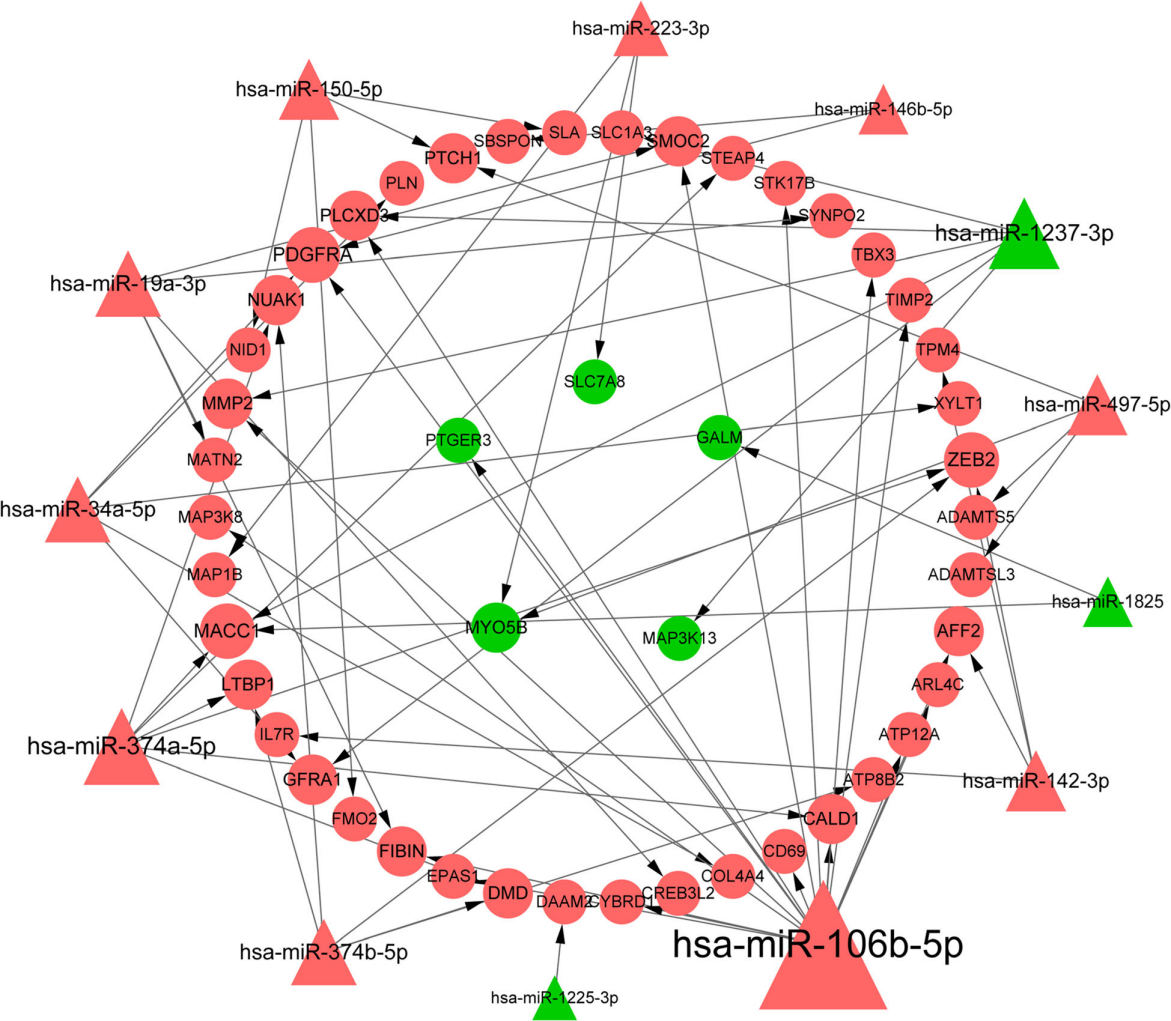
The miRNA-mRNA regulatory network. Triangles and circles indicate the miRNAs and genes, respectively. Orange and green color notes the upregulation and downregulation in diabetic samples versus control samples, respectively. The node size indicates the coordinated interaction degree

The miRNA-mRNA regulatory network was constructed to investigate the regulatory mechanisms involving the DEmiRNAs and DEGs in DN. Before the prediction of miRNA targets, 10 upregulated and 10 downregulated DEGs with the top absolute values of log_2_FC were selected (Table 1). Then, a total of 47 DEGs (42 up- and 5 downregulated DEGs) were identified to be targeted by 13 DEmiRNAs, including 10 upregulated DEmiRNAs and 3 downregulated DEmiRNAs. Subsequently, the differentially expressed miRNA-mRNA network was constructed (Fig. 4). In this network, 17, 7, and 6 DEGs were targeted by *hsa-miR-106b-5p*, *hsa-miR-374a-5p*, and *hsa-miR-1237-3p*, respectively.

**Table 1:** The list of the top 20 differentially differentially expressed miRNAs in diabetes versus controls

miRNA	logFC	*P*.Value	adj. *P*.Value
**Top 10 downregulated miRNAs**			
hsa-miR-223-3p	4.55	8.49 × 10^−7^	9.77 × 10^−5^
hsa-miR-150-5p	4.42	7.13 × 10^−5^	1.07 × 10^− 3^
hsa-miR-142-3p	3.88	2.26 × 10^−5^	5.27 × 10^− 4^
hsa-miR-34a-5p	3.79	1.22 × 10^−6^	1.20 × 10^− 4^
hsa-miR-19a-3p	3.69	1.12 × 10^−5^	3.42 × 10^−4^
hsa-miR-374b-5p	3.65	9.34 × 10^−8^	2.60 × 10^−5^
hsa-miR-146b-5p	3.58	1.35 × 10^−8^	1.19 × 10^−5^
hsa-miR-106b-5p	3.42	6.17 × 10^−6^	2.77 × 10^−4^
hsa-miR-374a-5p	3.40	1.35 × 10^−6^	1.20 × 10^−4^
hsa-miR-497-5p	3.35	2.14 × 10^−5^	5.13 × 10^−4^
**Top 10 upregulated miRNAs**			
hsa-miR-1237-3p	−3.02	3.88 × 10^−4^	3.38 × 10^− 3^
hsa-miR-1973	−3.25	6.97 × 10^− 3^	2.42 × 10^− 2^
hsa-miR-1281	− 3.31	1.26 × 10^− 4^	1.70 × 10^− 3^
hsa-miR-1225-3p	− 3.31	1.46 × 10^− 4^	1.83 × 10^− 3^
hsa-miR-425-3p	− 3.47	1.60 × 10^− 4^	1.92 × 10^− 3^
hsa-miR-1825	− 3.56	1.60 × 10^− 4^	1.92 × 10^− 3^
hsa-miR-1234	−3.57	5.06 × 10^−5^	9.08 × 10^− 4^
hsa-miR-1238	−3.58	6.02 × 10^−5^	1.03 × 10^− 3^
hsa-miR-191-3p	−3.59	8.00 × 10^−5^	1.16 × 10^− 3^
hsa-miR-1228-3p	−3.61	3.95 × 10^−5^	7.62 × 10^−4^

### Construction of the lncRNA-miRNA-mRNA regulatory network

**Fig. 5:**
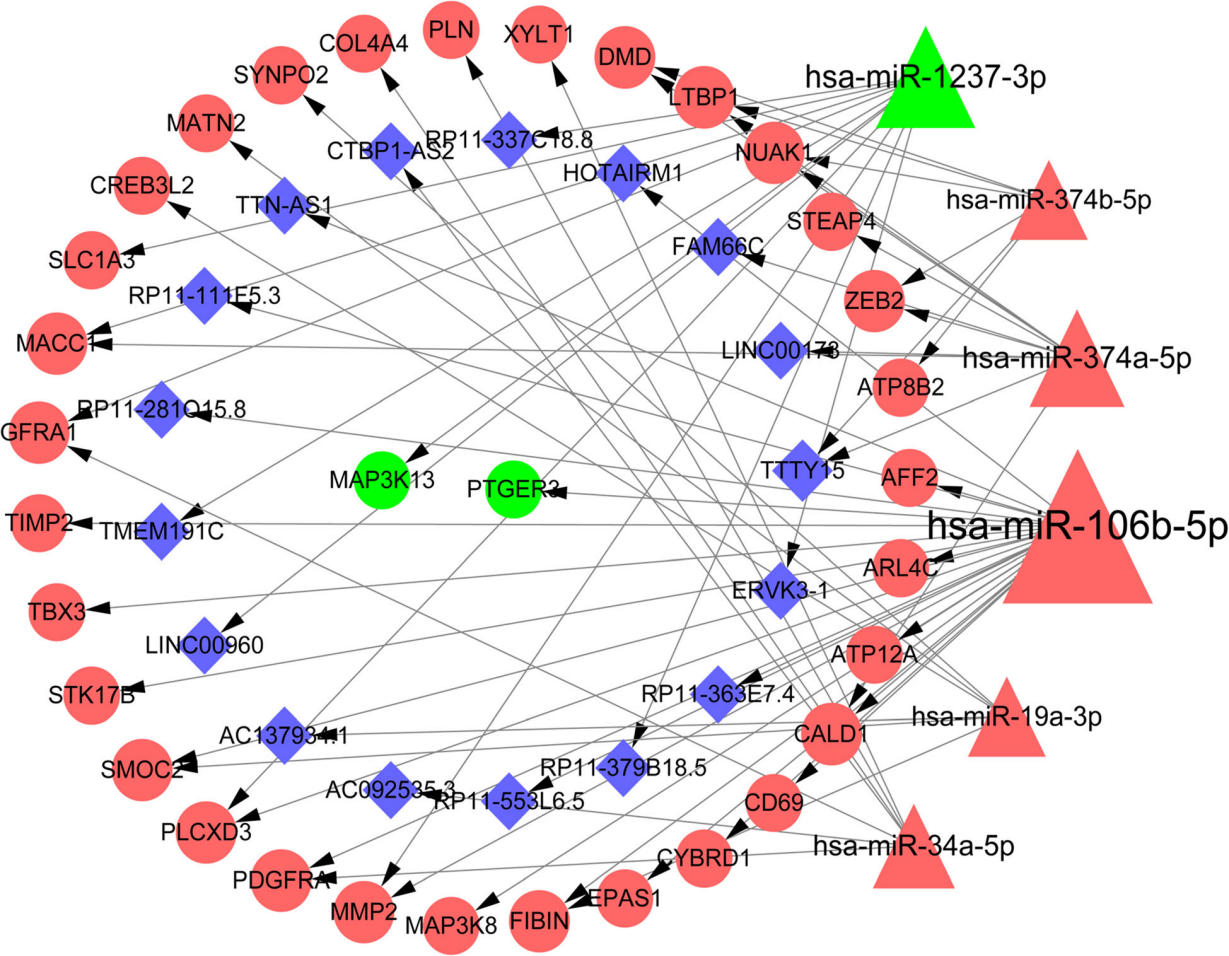
The lncRNA-miRNA-mRNA regulatory network. Diamonds, triangles and circles indicate the lncRNAs, miRNAs and genes, respectively. Orange and green color notes the upregulation and downregulation in diabetic samples versus control samples, respectively. The node size indicates the coordinated interaction degree

The ceRNA network was constructed using the 6 miRNAs had interaction degrees of greater than 5 in the miRNA-mRNA network, including *hsa-miR-106b-5p*, *hsa-miR-374a-5p*, *hsa-miR-1237-3p*, *hsa-miR-19a-3p*, *hsa-miR-34a-5p*, and *hsa-miR-374b-5p*. Eighteen lncRNA-miRNA pairs had scores of higher than 0.99 were screened out accordingly. After integrating the aforementioned miRNA-mRNA and lncRNA-miRNA pairs, 63 lncRNA-miRNA-mRNA pairs were screened out. The corresponding ceRNA network was composed of 63 lncRNA-miRNA-mRNA pairs, 6 DEmiRNAs, 33 DEGs, and 17 lncRNAs (Fig. 5). In this network, *hsa-miR-106b-5p*, *hsa-miR-1237-3p*, and *hsa-miR-374a-5p* had a relative higher interaction degree of 23, 11 and 10, respectively. The potential ceRNA networks included *RP11-363E7.4*/*TTN-AS1*/*HOTAIRM1*-*hsa-miR-106b-5p*-*PTGER3* and *LINC00960*-*hsa*-*miR*-*1237*-*3p*-*MMP*-2.

### Identification of potential drug targets

**Fig. 6:**
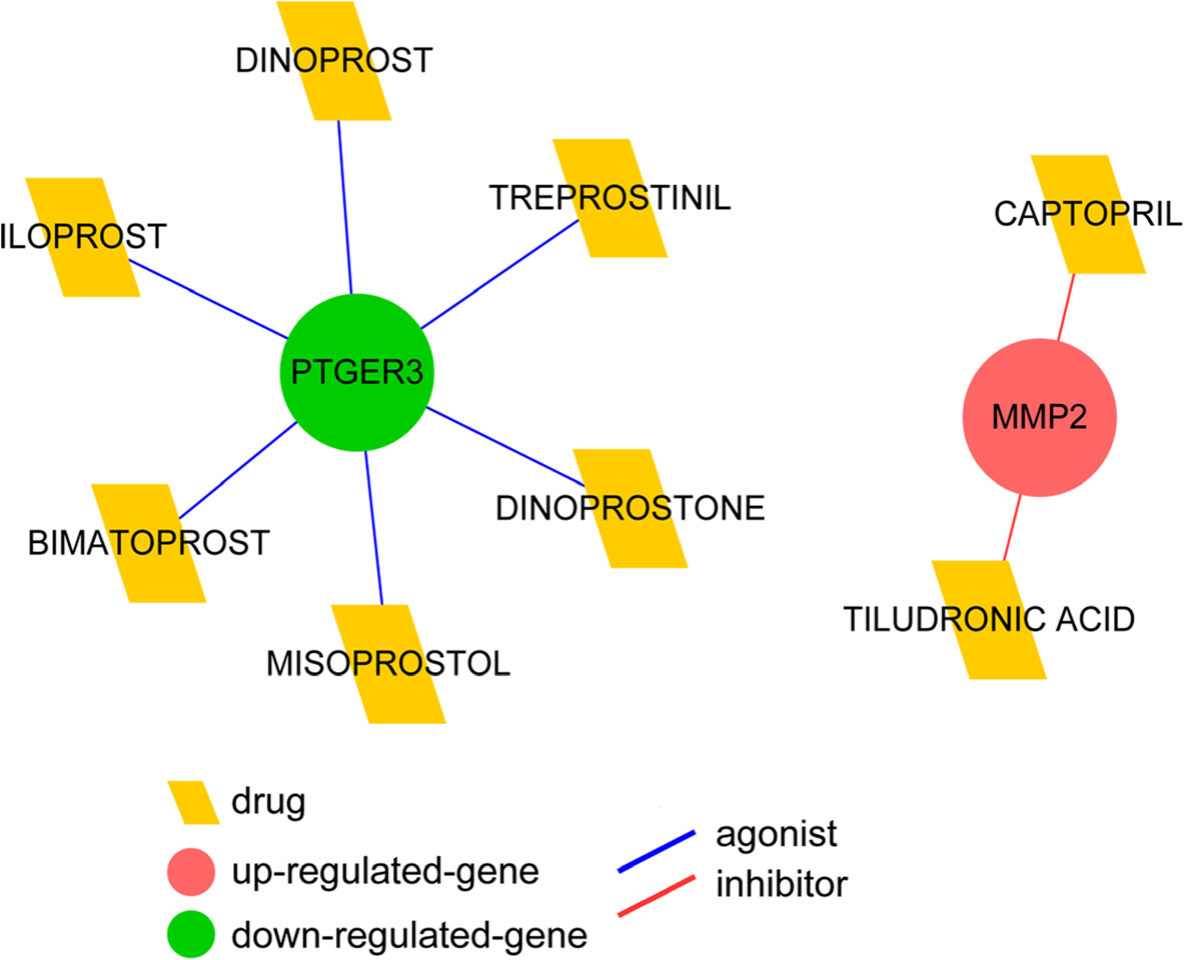
The drug and gene interaction network involving two key candidates

To understand the potential drugs targeting *PTGER3* and *MMP*-2, the drug-gene interactions were predicted and the gene-drug interaction network involving the two genes was constructed (Fig. 6). It was composed of 6 *PTGER3* agonists (bimatoprost, dinoprostone, misoprostol, iloprost, dinoprost, and treprostinil) and two *MMP-2* inhibitors (captopril and tiludronate; Fig. 6).

### Validation of the *MMP-2* and *PTGER3* expression

**Fig. 7:**
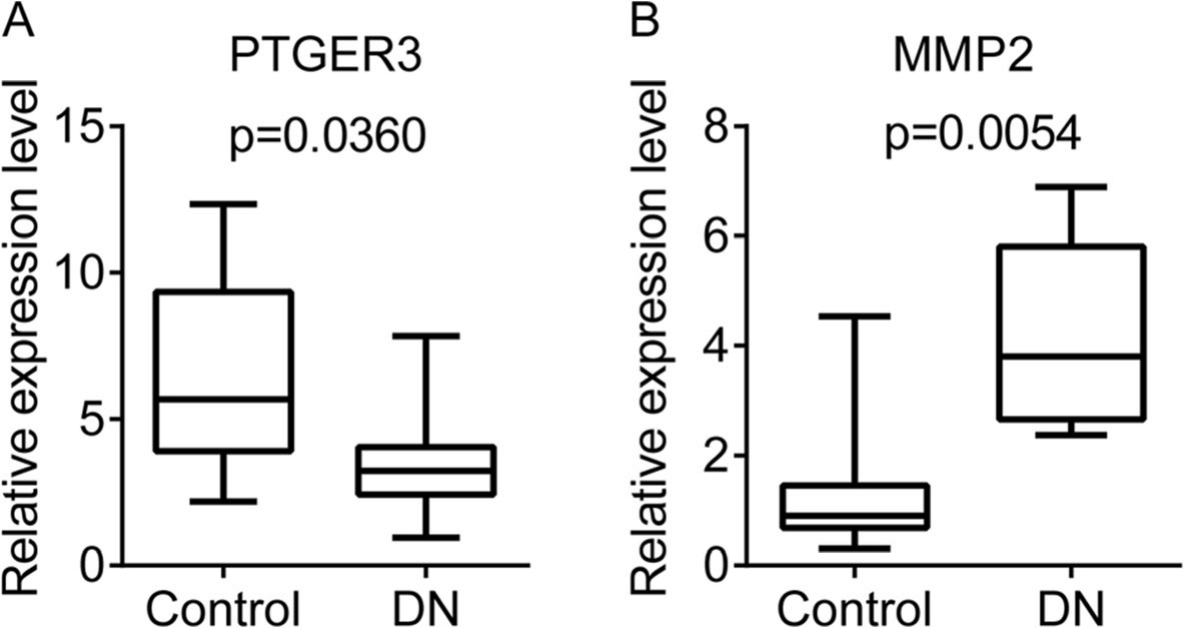
The expression of the two genes in the plasma samples from patients with diabetic nephropathy (DN). **a** and **b**, the expression levels of the *PTGER3* gene and the *MMP-2* gene in the plasma samples from patients with DN, respectively. The difference was analyzed using the t-test

The PCR analysis showed that there were significant differences in the expression levels of the *MMP-2* and *PTGER3* genes in the plasma samples between patients with DN and healthy controls (Fig. 7a and b). The expression level of the *PTGER3* gene in patients with DN was significantly lower than that from healthy controls (*p* = 0.0360, Fig. 7a), while the *MMP-2* gene was significantly upregulated in DN patients as compared with controls (*p* = 0.0054, Fig. 7b).

## Discussion

Based on the integrated bioinformatics analysis using GSE51674 and GSE111154 datasets, we finally identified two potential therapeutic targets in DN, including downregulated *PTGER3* and *hsa-miR-1237-3p*, upregulated *MMP-2* and *hsa-miR-106b-5p*. In addition, ceRNA interactions including *RP11-363E7.4*/*TTN-AS1*/*HOTAIRM1*-*hsa-miR-106b-5p*-*PTGER3* and *LINC00960*-*hsa-miR-1237-3p*-*MMP*-2 might be of novel and great value for clearing the pathogenesis and progression of DN. We also identified the therapeutic possibilities of targeting *PTGER3* and *MMP*-2.

The member of the EP receptor family, prostaglandin EP3 receptor gene (*PTGER3*/EP3) is a G protein-coupled receptor that facilitates a broad range of physiological actions including blunting glucose-stimulated insulin secretion (GSIS) [21]. It has been linked to the dysfunction and loss of β-cells in type 2 DM [21, 22]. *PTGER3* upregulation in the islets of patients with type 2 DM has been proven previously [23]. Additionally, elevated production of prostaglandin E_2_ (PE2) and PTGER3 can be targeted to improve insulin secretion from islets [24]. Pathologically, *PTGER3* negatively regulates the production of cyclic AMP (cAMP), a potentiator of GSIS [22, 25]. Rankin et al. [21] showed that agonist (sulprostone) and antagonist of EP3r both significantly reduced GSIS and fasting plasma glucagon in non-human primates with noninsulin-dependent diabetes mellitus. Schaid et al. [24] showed that γPTGER3 was constitutively activated in pancreatic β-cells and its action of reducing insulin was Gαz dependent. The αPTGER3 activity, however, could be stimulated by sulprostone in Gαz/cAMP-dependent and -independent signalings. After treatment with sulprostone, Rap1GAP-mediated inhibition of GSIS was potentiated in β-cells [24]. This was in line with the results reported by Rankin et al. [21].

However, the association of *PTGER3* expression with nephropathy or other renal diseases is rarely reported [3, 26, 27]. In renal cell carcinoma (RCC), the lower expression of *PTGER3* was correlated with a worse prognosis [26]. In patients with DN, downregulation of *PTGER3* in the kidney from donors with early DN versus matched controls has been confirmed by Sircar et al. [3]. What’s more, there was a differentially methylated level in the *PTGER3* gene between diabetic patients with end-stage renal disease and those without nephropathy [27]. Two or more methylated CpG sites were found in the *PTGER3* gene in diabetic patients with the end-stage renal disease compared with those without nephropathy [27]. An in vivo animal experiment showed that the knockout of *PTGER3* did not reduce the levels of plasma and urine glucose in the streptozotocin-induced mouse model of diabetes [28]. However, there was a significant increment in urine osmolality, water reabsorption, and aquaporins expression in mice kidney. Obvious reductions in renal hypertrophy, hyperfiltration, albuminuria, tubular dilation, and nuclear cysts were observed in the kidney in streptozotocin-treated *Ep*_*3*_^−/−^ mice compared with wild type mice [28]. These results showed that *PTGER3* downregulation was a specific protection for the kidney against metabolic disorders in diabetes. Its downregulation might be used as a biomarker or therapeutic strategy for DN.

Apart from the *PTGER3* gene, matrix metalloproteinase-2 (*MMP*-2) is also differentially methylated in diabetic patients with the end-stage renal disease compared with those without nephropathy [27]. MMP-2 is a well-characterized protein responsible for tissue remodeling and extracellular matrix degradation. *MMP*-2 expression is a marker of newly formed β-cells and is negative in the islets of adult rats [29]. It was positively correlated with periodontitis severity in patients with type 2 DM [30]. In addition, the expression of *MMP*-2 was higher in patients with type 2 DM compliant with the peripheral arterial disease compared with diabetic patients without arterial diseases and control groups [31, 32]. In patients with chronic kidney diseases, creatinine level was positively correlated with *MMP*-2 (r = 0.39), but in diabetic patients it was negative correlation (r = − 0.72) [31]. Our present study confirmed the upregulation of *MMP*-2 in DN might be associated with the remodeling of renal and preventing sclerosis in glomerular. The potential of employing if as a biomarker for DN in patients with diabetes should be validated.

Based on the analysis of DEmiRNAs in DN, we identified the potential regulatory networks in DN, including *RP11*-*363E7*.4/*TTN*-*AS1*/*HOTAIRM1*-*hsa*-*miR-106b-5p*-*PTGER3* and *LINC00960*-*hsa*-*miR-1237-3p*-*MMP-2*. In direct contrary to *PTGER3* and *MMP*-2, *hsa-miR-106b-5p* and *hsa-miR-1237-3p* were up- and down-regulated in DN versus control, respectively. *Hsa-miR-106b-5p* has been proven to contribute to β-cell proliferation following bone marrow transplantation [33]. It was also decreased in diabetic patients with chronic kidney disease versus patients without kidney disease [34], and is significantly linked to kidney function and eGFR [35]. *HOTAIRM1* expression is crucial for the differentiation of embryonic stem cells to renal lineage [36]. However, there is less information on the association of these miRNAs and lncRNAs with DN and the progression of other renal diseases. The tight interactions between these lncRNAs, DEmiRNAs (*hsa-miR-106b-5p* and *hsa-miR-1237-3p*), and DEGs (including *PTGER3* and *MMP*-2) indicated the potential and important roles of them in regulating kidney function.

Here in our study, we identified that *PTGER3* and *MMP*-2 were targeted by drugs including iloprost, treprostinil, and captopril. Iloprost and treprostinil are clinically used for severe pulmonary arterial hypertension and chronic thromboembolic pulmonary hypertension [37, 38]. Iloprost shows efficacy in decreasing urinary albumin excretion rate in patients with DN and attenuating the progression of DN [39]. Iloprost could downregulate *MMP*-2 via direct targeting [40]. Since the downregulation of *PTGER3* and upregulation of *MMP*-2 are protective for renal diseases, the agonists of PTGER3 (including iloprost and treprostinil) and inhibitor of MMP-2 (like captopril) might be inapplicable for the treatment of DN. Novel drugs aiming to increase *MMP*-2 or decrease *PTGER3* in the kidney might of great values for preventing DN and renal damages in diabetes.

## Conclusions

In summary, we confirmed the crucial roles of *PTGER3* downregulation and *MMP*-2 upregulation in the kidney from patients with DN. They were regulated by lncRNAs (including *RP11-363E7.4*, *TTN-AS1*, *HOTAIRM1*, and *LINC00960*) and miRNAs (including *hsa-miR-106b-5p* and *hsa-miR-1237-3p*) that had been rarely reported in DN. Their dysregulation might be the defense mechanism against renal injury following diabetes. However, more preclinical experiments and clinical trials should be performed to determine the dysregulation of them was a blessing or a curse for DN.

## Data Availability

The human miRNA microarray dataset GSE51674 and gene expression dataset GSE111154 are available at the National Center for Biotechnology Information (NCBI) Gene Expression Omnibus (GEO, http://www.ncbi.nlm.nih.gov/geo/). The original raw data for the PCR analysis in DN patients were available from the corresponding author with reasonable requirement.

## Abbreviations

DM: Diabetes mellitus
DN: Diabetic nephropathy
eGFR: Estimated glomerular filtration rate
GEO: Gene Expression Omnibus
DEGs: Differentially expressed genes
DEmiRNAs: Differentially expressed miRNAs
KEGG: Kyoto Encyclopedia of Genes and Genomes
PPI: Protein-protein interaction
ceRNA: Competing endogenous RNA
DGIdb: Drug-Gene Interaction database
FDA: Food and Drug Administration
GSIS: Glucose-stimulated insulin secretion
PE2: Prostaglandin E_2_
cAMP: Cyclic AMP
*PTGER3*: Prostaglandin EP3 receptor gene
RCC: Renal cell carcinoma
*MMP*-2: Matrix metalloproteinase-2
